# The journey of service users with complex mental health needs: a qualitative study

**DOI:** 10.1080/21642850.2024.2365226

**Published:** 2024-06-14

**Authors:** Laura Sambrook, Anna Balmer, Hana Roks, Jackie Tait, Peter Ashley-Mudie, Jason C. McIntyre, Amrith Shetty, Christopher Bu, Rajan Nathan, Pooja Saini

**Affiliations:** a Faculty of Health, Liverpool John Moores University, Liverpool, UK; b Cheshire and Wirral Partnership NHS Foundation Trust, Chester, UK

**Keywords:** Mental health, complex needs, qualitative research, health service research

## Abstract

**Background:** This study aimed to provide a robust picture of the journey of service users with complex mental health needs by evaluating the perspectives of service users and carers with lived experience of services and gaining clinician views about decision making in relation to this cohort.

**Methods:** A qualitative design was used. Service users (*n* = 11), carers (*n* = 10) and clinicians (*n* = 18) took part in semi-structured interviews, which were transcribed verbatim and analysed using thematic analysis.

**Results:** The following themes were identified by participants: ‘relationships with staff,’ ‘treatment options, pathways and availability,’ ‘the role of autonomy in recovery,’ ‘impact of out-of-area placements,’ and ‘specialist training for staff.’ The findings demonstrated that the journey of serviceusers can be impacted by a wide range of factors, including relationships with staff, the nature of support offered, community response, financial constraints, and organisational goals around bed pressures.

**Conclusions:** Recommendations include the need for staff to work in partnership with service users and carers, foster autonomy, access specialised suicide prevention training, and agree discharge and contingency plans with service users. Further work is needed to deliver the best possible experience for individuals with complex mental health needs and those who care for them.

## Introduction

One in four adults in England experience at least one diagnosable mental health (MH) condition in any given year (Mind, 2020), with NHS England (NHS England, 2019) stating that MH problems represent the biggest single cause of disability in the UK. There is a growing awareness that individuals with complex MH (CMH) needs are not best served by generic, community-based service delivery models, highlighting the need to examine their experiences of care. In complex cohorts, four out of five have a diagnosis of psychosis, severe negative symptoms, and cognitive impairments; many have coexisting MH problems and physical health concerns resulting from poor lifestyle conditions and side effects of psychotropic medications (Killaspy, 2014). The majority of this group have been in contact with MH services for many years and have experienced repeated acute psychiatric admissions or the use of rehabilitation services (Dalton-Locke et al., 2021; Killaspy & Zis, 2013).

When considering the service user journey, we must also include the voices of their carers and the clinicians working with them. As MH care is now increasingly provided in the community, loved ones of individuals with long-term MH problems are relied upon to provide daily support as informal, non-clinical carers (Stuart et al., 2020). In the UK alone, there are an estimated 6.8 million informal carers who, through their unpaid care, save the taxpayer approximately £132 billion annually (Abou-Seif et al., 2022). This can be debilitating for the carer themselves, resulting in mental and physical health difficulties (Barrow & Harrison, 2005), burnout (Stuart et al., 2020), financial burden (Lawn, 2015), and fear (Gray et al., 2010). Carers of those with diagnoses such as borderline personality disorder (BPD) can experience additional stressors, such as exposure to repeated self-harm or suicide attempts, intense anger from their loved one, and discrimination from their community, often due to a lack of understanding of the diagnosis (Lawn, 2015). Greater carer involvement in treatment is associated with improved outcomes; however, many carers report feeling excluded by MH services, and that their needs often go unrecognised (Stuart et al., 2020). Within the small body of published research, carers reported a desire for increased involvement in treatment decisions (Lawn, 2015), trusting relationships with clinicians (Stuart et al., 2020), and the provision of clear information about treatment plans (James, 2012).

As the needs of service users with complex diagnoses or comorbidities do not map readily onto existing structures of service provision (Rock & Carrington, 2012), MH teams can find it difficult to reach clinical decisions about their care. There is a sparsity of research into clinician decision-making (Kon, 2010); however, a variety of factors have been found to influence this more generally, including risk assessment (Muir-Cochrane et al., 2011), application of relevant knowledge (Miller et al., 2020), intuition (Magnavita, 2016), and emotional reasoning Nathan et al., 2021. Service user complexity can make this process more challenging, with qualitative research into clinician experiences of working with individuals with BPD emphasising that their work can be emotionally demanding (Bowen, 2013) and dangerous, due to increased risks such as patient aggression, substance misuse and self-harm (Muir-Cochrane et al., 2011). With this in mind, it is important to consider the experiences and perspectives of clinicians responsible for decision-making for this cohort, as well as how the decisions they make may impact the service user journey as a whole.

Individuals with CMH needs are often accommodated in out-of-area placements (OAPs) that are a long distance from their loved ones (Chinn et al., 2011), due to the inability of local services to meet their needs. In addition to being costly to the NHS and local social care authorities, individuals placed out-of-area can achieve poorer outcomes (Beadle-Brown et al., 2005), experience disruptions to their lives (Galante et al., 2019) and, in some cases, be over supported (Rambarran, 2013). As little regulation exists surrounding such placements, and because OAPs are viewed as a way to contain those that NHS services find troubling (Care Quality Commission, 2014), service user experience must be examined.

The present study aimed to evaluate the perspectives of service users and carers with lived experience of specialist placements, as well as their overall experiences of care, and learn more about the experiences of clinicians working with this group. Whilst our primary concern was to explore the perspectives of service users and carers, we felt our findings would be enhanced by also taking account of clinicians’ perspectives, as we felt this would provide further information about how these individuals come to be classified as ‘complex’ and the challenges clinicians may face when making decisions about their care. The research addresses a gap in the literature, as, to our knowledge, no qualitative studies to date have included triangulation data from service users, carers and clinicians to offer insight into the journey of service users with CMH needs and provide recommendations for improving future practice.

## Materials and methods

### Design

Semi-structured one-to-one interviews were conducted, exploring the experiences and perspectives of service users with CMH needs, carers, and clinicians, as part of a qualitative thematic analysis (Braun & Clarke, 2006).

### Study setting

Cheshire and Wirral Partnership NHS Foundation Trust (CWP) provide a range of community and inpatient physical and MH care services. The Trust also provide care to a specific cohort of service users with CMH needs. This is a broad term used to describe those who receive packages of care commissioned by NHS Cheshire CCG either in an inpatient or community setting. The OAPs are delivered by a range of other healthcare providers. This study mostly included service users who were detained under Section 17 of the Mental Health Act or Section 117 Aftercare, or who had learning disabilities, acquired brain injuries, or physical disabilities. Ethical approval was obtained from the NHS Health Research Authority and West Midlands – Coventry & Warwickshire Research Ethics Committee: Integrated Research Application System (IRAS) prior to study commencement [REC Ref: 21/WM/0020].

### Participants

Eleven service users, 10 carers and 18 clinicians were interviewed. It was a requirement of the study that service users were recorded by the Trust’s clinicians as having complex, long-term recovery needs and long-standing MH problems, or caring for someone with these issues. Clinicians needed to be employed by the Trust and involved in making clinical and pathway decisions for this cohort. Participants were excluded if they were under 18 or unable/unwilling to provide written informed consent.

### Materials

Participants were provided with a participant information sheet and consent form to sign prior to taking part. Interview schedules were developed for the semi-structured interviews (see supplementary materials), with input from relevant stakeholders comprising representatives of CWP, public and patient involvement, commissioners and local authority staff. Service user interview schedules covered topics such as diagnoses, placements and relationships with staff, with questions such as, ‘what are your thoughts about the care you have received for your MH condition?’ Carer interview schedules focused on the impact of these matters on the carer, for example, ‘can you tell me about your experience of caring for someone with CMH needs?’ The clinician interview schedules were asked in relation to a representative vignette.

Four vignettes were developed for the clinician interviews, illustrating four hypothetical clinical cases. Vignette one involved a man with a diagnosis of paranoid schizophrenia, who continued to have problematic use of drugs and non-compliance with inpatient procedures, resulting in an unsuccessful discharge from the rehabilitation ward. Vignette two focused on man with a diagnosis of paranoid schizophrenia, who had become institutionalised as a result of being under secure MH services for 20+ years; however, the rehabilitation team had expressed reservations about accepting his referral due to his high levels of disturbed behaviour. Vignette three involved a young woman with a multitude of diagnoses, who was on a rehabilitation ward and working towards discharge home but experiencing difficulties due to high levels of distress and self-harming behaviours. Vignette four focused on a man with diagnoses of schizoaffective disorder and autistic spectrum disorder (ASD) who had experienced a period of revolving door admissions; however, his discharge from inpatient care was proving difficult due to his history of violent and drug-taking behaviours. Topics discussed in the interviews included decision-making, support needed to improve decision-making processes, and alternative approaches for people who present with a similar clinical and risk profile to the service user in the vignette. Although the vignettes differed for each clinician interview, a standard set of questions were used to start the discussion.

### Procedures

**Figure 1.**
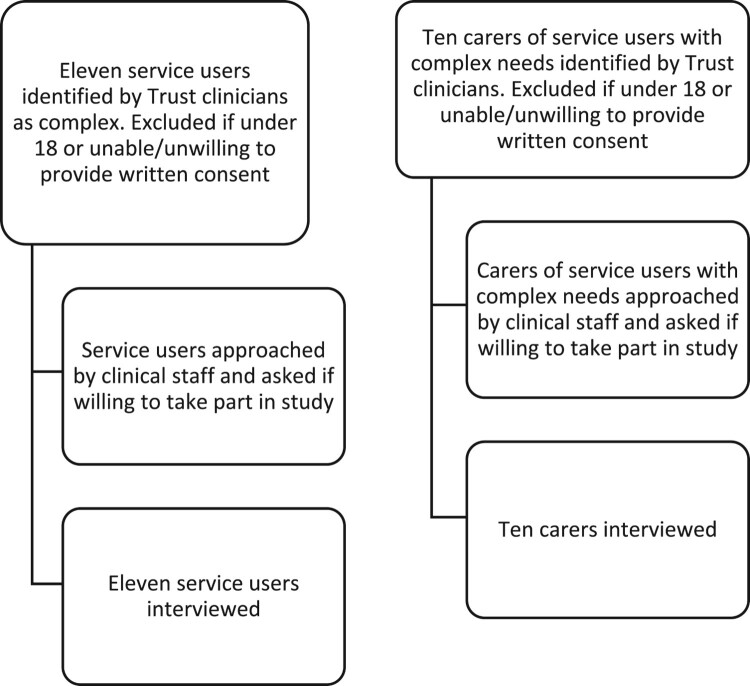
Recruitment process for service users and carers.

**Figure 2.**
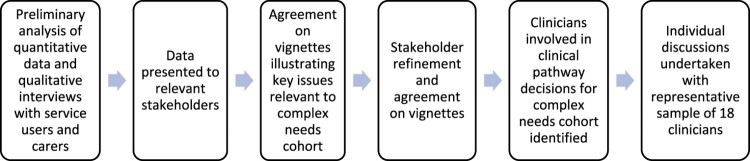
Recruitment process for clinicians.

Service users and carers were identified by Trust clinicians as suitable to participate in the study, then approached by a member of the research team (see Figure 1). For clinician recruitment, a member of the research team (TN) identified 29 clinicians, from various professional backgrounds, with involvement in making clinical decisions for this cohort. They were approached via email, with 18 agreeing to take part (see Figure 2). For those who consented, a date was agreed for a remote interview to take place, due to ongoing COVID-19 restrictions.

### Data analysis

With the participant’s permission, discussions were recorded, transcribed verbatim by ‘UK Transcription,’ checked against the audio files for accuracy by the researchers who conducted the interviews (LS, AB, HR), and analysed using thematic analysis based on Braun and Clarke’s six-stage process (Braun & Clarke, 2006; Braun & Clarke, 2019), due to its flexibility and potential to deliver rich and complex understandings. All transcripts were analysed by eight members of the research team, each with different disciplinary backgrounds (PS, TN, CB, LS, AB, HR, JT and PAM). The data were coded using NVivo software, developed into an unrefined map of codes and themes, and further refined through continued reading and analysis in an iterative process. The iterative coding process enabled the continual revision of themes until the final classifications of major themes were agreed. During repeated rounds, frequent comparisons were made across codes and the interview data to develop, review, and refine themes based on the complementarity, convergence, and dissonance of ideas across data sources (Braun & Clarke, 2006; Farmer et al., 2006). Themes were identified at a semantic level, recognising concepts directly communicated by participants, although consideration was given to possible deeper, latent concepts. To establish procedural reliability and conceptual credibility (Leung, 2015), additional members of the research team with experience in qualitative methods examined a sample of transcripts to compare their perceptions of the interview data and analysis with the main analyst’s interpretation. All findings were critically tested within the research group and disagreements were resolved by discussion.

## Results

The service users predominantly identified as male (*n* = 7) and were from White British backgrounds (*n* = 10). Seven service users were inpatients and four were living in the community (see Table 1). The carers and clinicians primarily identified as female (*n* = 7 and *n* = 11, respectively). The clinicians’ average length of time in their current post varied from 1 month to 8 years, although most had previous experience of working within the Trust (see Table 2).

**Table 1.** Service user and carer demographics.

Pseudonym	Gender	Ethnicity	Placement	Experience of OAP	Duration of interview
***Service User***					
Chloe	Female	White British	Community	Yes	79 min
Sarah	Female	White British	Inpatient	Yes	33 min
Sam	Male	White British	Inpatient	Yes	66 min
Eric	Male	White British	Inpatient	No	15 min
Jacob	Male	White British	Community	Yes	65 min
Micah	Male	White British	Inpatient	Yes	31 min
Chelsea	Female	White British	Inpatient	No	20 min
Matthew	Male	White British	Community	Yes	50 min
Catherine	Female	White British	Community	Yes	21 min
Trevor	Male	White British	Inpatient	Yes	32 min
Alex	Male	White Asian	Inpatient	No	45 min
***Carer***					
Diana	Female	n/a	n/a	Yes	17 min
Ruth	Female	n/a	n/a	Yes	58 min
Christine	Female	n/a	n/a	Yes	22 min
Ruby	Female	n/a	n/a	Yes	30 min
Amanda	Female	n/a	n/a	Yes	62 min
Michael	Male	n/a	n/a	No	37 min
Betty	Female	n/a	n/a	No	42 min
Andrea	Female	n/a	n/a	No	28 min
Peter	Male	n/a	n/a	No	32 min
Percy	Male	n/a	n/a	No	28 min

**Table 2.** Clinician demographics and roles.

Pseudonym	Gender	Age	Role	Time in Current Role	Professional Background	Duration of Interview
Paul	Male	nk	Psychiatry Consultant	nk	Psychiatry	53 min
Beatrice	Female	43 years	Psychiatry Consultant	4 years	Psychiatry	53 min
Sandra	Female	45 years	Psychiatry Consultant	6 years	Psychiatry	42 min
Randall	Male	47 years	Psychiatry Consultant	8 years	Psychiatry	36 min
Lauren	Female	27 years	Specialist Occupational Therapist	10 months	Occupational Therapist	45 min
Miguel	Male	nk	Psychiatry Consultant	nk	Psychiatry	38 min
Martin	Male	41 years	Consultant Social Worker	16 months	Social Work	49 min
Caroline	Female	44 years	Principal Clinical Psychologist	18 months	Clinical Psychology	47 min
Julie	Female	34 years	Clinical Lead	4 months	Clinical	33 min
Susie	Female	34 years	Principal Clinical Psychologist	17 months	Clinical Psychology	48 min
Sally	Female	31 years	Early Intervention Practitioner	1 month	Mental Health Nursing	39 min
Sabine	Female	32 years	Service Development Manager	3 months	Nursing	47 min
Laura	Female	50 years	Principal Clinical Psychologist	17 months	Clinical Psychology	38 min
Adam	Male	48 years	Staff Nurse	6 years	Mental Health Nursing	39 min
Rosie	Female	38 years	Clinical Lead	2 years	Mental Health Nursing	42 min
Marcus	Male	45 years	Advanced Nurse Practitioner	2 years	Nursing	27 min
Lydia	Female	33 years	Clinical Lead	1 year	Mental Health Nursing	44 min
Miles	Male	49 years	Nurse Consultant	9 months	Mental Health Nursing	46 min

**Figure 3.**
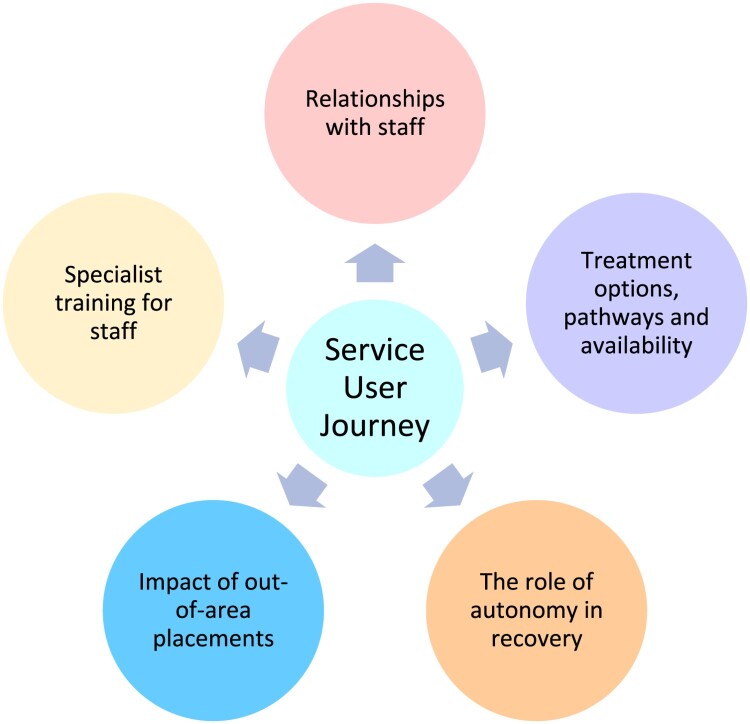
Service user journey themes diagram.

Five key themes were conceptualised regarding care for people with CMH needs (see Figure 3 and supplementary materials). All participant quotes are accompanied by a pseudonym and identified as service user (SU), carer (CA) or clinician (CL).

### Relationships with staff

#### The importance of strong therapeutic relationships

Positive interactions with staff members, including psychiatrists, nurses and psychologists, were reported. A common theme was the willingness of staff to make time for service users, even in the simplest of ways, such as asking them about their day, displaying genuine interest in their lives and comforting them when they were upset. Participants reported factors they believed to be indicative of a strong therapeutic relationship, such as mutual respect, honesty and transparency. Co-production between service users and clinicians had a positive impact both on service user outcomes and clinicians’ confidence in their decision-making, particularly in respect of inclusion in treatment decisions. Unhelpful experiences with staff were discussed, for example, feeling hurt if staff neglected to follow through with agreed plans; however, positive differences were reported when staff were honest and respectful:

I was fully informed about what I was taking and why I should be taking it. It felt like they had my best interests in mind. – Chloe (SU).

Clinicians recognised the importance of building strong therapeutic relationships, citing mutual respect and trust as key factors:

I remember this gentleman saying he works better with people when he feels they respect him. So they respect him and he respects them back. […] We formed that kind of relationship, and it did help because I listened to what he was saying and, equally, he listened to what I was saying. – Lauren (CL).

Carers highlighted the need for consistency when building therapeutic relationships, although this proved difficult during times of high staff turnover. Carers spoke of their loved ones’ pain when trusted staff members left and often watched their symptoms deteriorate, with some service users refusing to engage with new teams:

When people are suffering from mental health issues, they need to have somebody that is there for them, not 27 people who don’t have a clue who she is. […] To get the therapies into place, you need to build up a therapeutic relationship. – Amanda (CA).

#### Disparity in staff attitudes

An underlying theme was the expectation participants held about what a healthcare professional *should* be like, with some falling short of their expectations. Matthew (SU) felt staff were “just there for the wages” and Jacob (SU) spoke of staff presenting as uninterested and unengaged. Participants appeared to believe that staff should be naturally caring, and it was interactions with those without an empathic nature they found distressing. Participants considered environmental factors that may have influenced negative staff behaviours or impacted job performance, by contemplating the emotional impact of working in healthcare:

I think staff who are quite … I don’t want to say rude, but I think you may know what I mean. I think that’s due to them being so burnt out and they don’t have the capability to be compassionate all the time because it’s so draining for them. – Jacob (SU).

The staff […] work incredibly hard under huge pressure. – Andrea (CA).

Clinicians provided insight into how burnout can manifest when working with individuals with CMH needs, suggesting they attributed burnout to managing difficult service users rather than the demands of the role:

It’s very common for us to default back to our, I guess, basic settings, which is arriving at fairly quick and moral value judgements and say that this is just somebody who is challenging or problematic or manipulative. – Miguel (CL).

### Treatment options, pathways and availability

#### Diagnosis

The impact of receiving a MH diagnosis was a topic that divided the participants, with some stressing the importance of a diagnosis in relation to their identity, and others discussing the struggle of not agreeing with their diagnosis or feeling they had been treated differently because of it:

For a long time in the community, they were like, ‘personality disorder,’ but they did the proper personality disorder questionnaire, and I don’t have one, so that meant I was diagnosed with bipolar disorder. Then, after that diagnosis, I got the right treatment, so the diagnosis was important. – Chelsea (SU).

Variation existed amongst the clinicians about the importance of a MH diagnosis; some expressed that positive treatment outcomes could not be achieved without the correct diagnosis, whilst others viewed a diagnosis as one aspect of a much broader picture. The clinicians discussed how multiple diagnoses could make decision-making around future interventions and placements more difficult:

The number of diagnoses is very unhelpful. […] I think it happens a lot. I’ll often meet somebody and they’ve got an array and we’re not sure what to focus on. – Caroline (CL).

Several participants reflected on whether or not a diagnosis was helpful in terms of their sense of self:

I can understand why someone might want a diagnosis. It’s comforting to know you haven’t made everything up. It gives you a sense of identity. – Jacob (SU).

Do you label people or don’t you? […] If you get labelled too soon, you try to become that label. – Christine (CA).

Although diagnostic fluidity may be beneficial in practical terms, the discussions highlighted that it may be helpful for clinicians to consider the potential impact on the individual and their family when issuing or changing a diagnosis.

#### Interventions

The need for early intervention was highlighted by seven participants. Some expressed frustration at how their behaviour as children should have been acknowledged as an indicator of a need for additional support; however, many felt invalidated by the excuses people made for their actions:

I was mentally disturbed from a young age at primary school, at secondary school, and it never really got picked up on. […] No one ever questioned why I was behaving the way I was behaving; I was just treated as a young offender. – Sam (SU).

Some believed they could have recovered more quickly, or experienced less severe symptoms, if they had been offered an intervention earlier, with carers reporting similarly:

In the community, I was ill for about three, four years before I had the psychotic episode. Early intervention would definitely have helped, if they had taken it seriously. – Chelsea (SU).

It was only when she got to the point where she tried to kill herself that people took her seriously. When a young person asks for help, they need to be treated there and then, not told, ‘we’ve got a really high demand, and we’ll see you in six months.’ By six months, that family is in crisis, or that young person has killed themselves. – Amanda (CA).

Considerable benefits of engaging with therapy were reported:

I felt the schema therapy was really suited towards my bipolar condition, because it went over things that happened when I was psychotic, which I felt ashamed about. It kind of rewired my brain into thinking less shame and guilt. – Chelsea (SU).

For some, engaging with psychological therapies led to a breakthrough and the beginnings of recovery. For others, it simply resulted in feeling more able to communicate their needs, de-escalate their symptoms, and problem solve. Despite this, some participants wished that support was more readily available, particularly trauma therapy. Service users mentioned other issues contributing to the failure of psychological interventions, including high staff turnover and a lack of specialised training:

Whenever you get a psychologist, they always end up leaving after so long and you’re never able to finish any piece of work, which is really debilitating. – Jacob (SU).

### The role of autonomy in recovery

#### Involvement in treatment decisions

Service user involvement in treatment decisions appeared to differ depending on placement. Some recalled having a high level of involvement in their care and feeling their voice had been heard concerning matters such as medication changes, care plan reviews and ward leave, resulting in a sense of validation. Some carers had positive experiences and felt they were given a voice in terms of the care of their loved one. Increased autonomy elicited positive feedback, highlighting the importance of joint working:

I always had the final say in my medication. Again, with the therapy, I had a lot of say in what kind of therapy I wanted. – Chelsea (SU).

Four clinicians discussed the importance of allowing service users to play an active role in the creation of their own treatment plans, and that this was their preference when decision-making:

I tend to see it as, ‘it’s not me who’s driving it. I want you to drive it.’ Most people in this situation feel out of control in their lives. They feel everyone else is making decisions for them. – Sandra (CL).

Despite this, the majority of discussions around autonomy highlighted the lack of input service users had in respect of their care. This was apparent in terms of medication, where service users reported a complete lack of involvement in most cases, which had a negative impact on therapeutic relationships:

They didn’t give me a choice. They basically said, ‘you’ve got to take this. If you don’t take it, then we’ll put you on a depot. Even if you refuse a depot, you’re still going to have it,’ so it was just forced, which made me feel more against the staff. – Chloe (SU).

Carers felt excluded from discussions; expressing their frustration at being ignored when they felt they could have provided valuable input:

There were so many times when I said, ‘I’ve been with her since she was born, and she’s happy for me to talk to you about whatever,’ but that was never really taken up. – Andrea (CA).

Often families get ignored in services and plans. – Caroline (CL).

#### Lack of person-centred care

Service users expressed a desire for treatment decisions to be considered on a person-to-person basis, as opposed to a ‘one size fits all’ approach. Some voiced their frustration about being pigeon-holed as offenders throughout their treatment journey:

They’re keeping people under sections when they’re showing no symptoms of psychosis. They’re exhibiting no risk to self or others, and they haven’t for over a year. – Alex (SU).

One clinician highlighted the importance of treating service users as individuals, especially for those with CMH needs, due to the increased likelihood of co-morbidities and specific requirements:

Not everyone fits into the same box, so we need to understand that person and see what services can be moulded around them. – Lauren (CL).

### Impact of out-of-area placements

Eight service users were placed away from their home communities during their treatment journeys, as local placements offering specialised support had not been available. The most notable impact was on their social lives, as their loved ones had to travel considerable distances to visit them:

It used to get me down sometimes because I’d only see my family like once a week […] and I used to see them all the time. – Trevor (SU).

Two service users reported that they would rarely receive visits due to the time and cost required for the journey. Carer discussions focused on the logistics of travelling to OAPs, such as time constraints, work responsibilities and a lack of funds:

It’s a 10-hour round trip, a big drive up. I mean, luckily I can drive, but I put the petrol in my car, I take up the stuff for her, and there is no help with regard to that. – Amanda (CA).

Only three clinicians viewed OAPs as a viable treatment option, reporting that sending an individual away can be beneficial if it means they will receive specialist care that their local Trust is unable to offer. The remaining nine clinicians highlighted why they can be detrimental:

I always find it is done with an ulterior motive. […] It is easy to transfer a patient and then it is somebody else’s problem, and you can almost forget it. In my experience, I always prefer to keep patients within the footprint of the home, of the Trust. – Marcus (CL).

The participants made recommendations about how to make the transition from a local placement to an OAP easier. Chloe (SU) had been abruptly moved and reflected on how this situation would have been less distressing had she been notified about the move in advance. Jacob (SU) had a further recommendation about creating a sense of familiarity within an OAP, which was strengthened by Ruby’s (CA) experience of watching her loved one deteriorate without familiar faces caring for her:

If you get moved and you’re very close with your CPN or your psychiatrist, even if they were able to have catch ups with you whilst you were at that unit. […] It’s being able to have that familiarity within that area, which might feel quite desolate. – Jacob (SU).

I think she became more institutionalised there and she deteriorated, and quite possibly it was the move to a new area where she didn’t know anybody and felt isolated. – Ruby (CA).

### Specialist training for staff

Training needs were reported for staff working with people with CMH who may present with suicidal behaviours and diagnoses including BPD. Four service users recalled experiencing suicidal thoughts and five carers discussed their loved one harming themselves. Some service users failed to understand why they had been discharged back into the community when they were still suicidal, as they felt this led to further suicide attempts and readmissions:

I was still actively suicidal, actively hating myself, actively doing stuff; things that were quite a danger to myself. And they still discharged me, because they said it would be more beneficial to be in the community. […] At that time, I was not ready to be discharged. – Sarah (SU).

Between 2009 and 2019, there were 5218 (29%) patients who died by suicide in acute care settings; the majority (84%) were viewed by clinicians as presenting with low or no short-term risk (The National Confidential Inquiry into Suicide and Safety in Mental Health, 2022). Participants reported feeling that sudden discharge placed unnecessary stress onto everyone involved, and the consensus was that staff would benefit from additional training into supporting individuals to reintegrate back into the community using a gradual approach to promote relapse prevention:

Gradually reintroducing me back into the community was really helpful. Rather than saying, ‘you have all this leave,’ I was started off on a small amount and then it gradually increased. – Chelsea (SU).

The clinicians provided insight into working with high-risk service users, explaining how some will tolerate risk to support service users in working towards their long-term goals, whilst others are more risk-averse in their decision-making and prefer to follow restrictive practices to keep service users safe in the short-term:

We’ve got people in the team that have recently had a couple of suicides, they’ve got to do the coroner’s report. That’s going to affect their practice, that’s going to make their risk threshold a lot lower …  – Julie (CL).

Participants reported that staff stigma was associated with some MH conditions, particularly BPD. Service users explained that it was common for staff to treat them differently due to their diagnosis, which they felt impacted their overall experiences of care. Clinicians and carers reported similar feedback:

Because of some of my diagnoses, there’s a lot of stigma towards them and I think it’s a stigma that’s held from all levels of professionals. It’s held from support workers, nurses, occupational therapists, psychiatrists, psychologists. – Jacob (SU).

She came out with a diagnosis of EUPD. […] We’ve since found out the professionals don’t really know how to treat it. – Ruth (CA).

Some of the language, particularly to describe personality disorder, is really negative, inaccurate, and not clinically informed. – Lauren (CL).

Specialised staff training into personality disorders and how to treat them was recommended by participants, as they believed the stigma experienced was due to a lack of understanding.

## Discussion

### Main findings

We found that many participants had, at times, experienced excellent care. They highlighted positive interactions with staff who were empathic, spoke of the importance of being involved in treatment decisions and shared how access to therapy had aided their recovery. Mutual respect and co-production were valued by all three participant groups and appeared to be a common link between all themes, as therapeutic alliances, successful placements, increased autonomy and positive therapeutic outcomes could not occur without these elements in place. Similar findings have been reported for positive aspects of MH placements (Beadle-Brown et al., 2005; Chinn et al., 2011) with simple gestures of kindness such as an unplanned call or a hand to hold, which may be regarded as trivial in a professional context, can make a real difference to someone in crisis (Klevan et al., 2017). Less positive experiences were reported where service users felt they had been failed, with discussions centred around difficult encounters with staff, a lack of autonomy, stigma associated with their diagnosis and being placed away from home. Memories of unkind treatment by staff were upsetting for service users to recall, with clinicians attributing this to staff burnout associated with complex diagnoses (Bowers, 2005). The clinicians appeared to value their work with this service user group and recognised areas in which they could improve, highlighting how input from service users and an agreed mental health diagnosis increases their confidence in their decision-making. They also admitted that their ability to house service users in suitable placements is affected by the lack of local provision, and that working with high-risk service users can result in them engaging with risk-averse and overly restrictive practices.

A key finding of this study was the importance of person-centred care and autonomy, particularly with regards to recovery (Krotofil et al., 2018), and that lower levels of involvement led to disengagement and withdrawal. Preference relating to diagnosis and therapeutic options further evidenced the need for person-centred care. Another key finding related to the need for increased staff training into supporting service users and their families post-discharge. All participants agreed that organisational pressures can result in discharge prior to recovery, therefore it may be beneficial for future quantitative research to be conducted in this area involving outcomes and readmission data, to be considered alongside the NCISH (The National Confidential Inquiry into Suicide and Safety in Mental Health, 2022) findings. All participants stressed the need for ongoing support following discharge, such as phased discharge, signposting to community services and home visits.

### Comparison with other studies

Similarly to previous research (Gray et al., 2010; Lawn, 2015), carers discussed the impact of caring for someone with CMH needs on their own wellbeing. Distressing instances were reported whereby their voices were not heard by staff, which, in some cases, were perceived to result in self-harm, suicide attempts and repeated hospital admissions. Consistent with research into marginalisation of carers (Stuart et al., 2020), clinicians in our study recognised that carers can be excluded by MH services. Whilst the available evidence supports the benefit of carer involvement (Maybery et al., 2021), the findings suggest that there may be barriers to the routine implementation of this approach, including the involvement of informal carers. Further research would need to identify the reasons, but clinical experience would suggest that possible factors may include limited awareness of the evidence supporting the effectiveness of carer involvement, pressure on time and resource, and a narrow medical focus on the condition (rather than the person as a whole and their life circumstances). Carer recommendations included a desire for increased involvement in treatment decisions (Lawn, 2015), strong therapeutic relationships with clinicians (Stuart et al., 2020), early intervention in schools and increased support following discharge into the community.

Service users reported social dislocation, low mood and reduced contact with loved ones as a consequence of being placed away from their home communities, with family members forced to drive considerable distances to visit. Similar findings were reported (Chinn et al., 2011; Rambarran, 2013; Ryan et al., 2004) on how this affected carers in respect of childcare, work responsibilities and funds. Although some participants cited benefits of OAPs, such as focusing on treatment without distractions and accessing improved specialist care, the consensus was that service users who had been admitted to an OAP were more likely to struggle to reintegrate back into their home communities, due to poor communication between services and disengagement. The consensus was that specialist services should be provided locally wherever possible, with OAPs used only when individuals cannot be treated locally for reasons such as experiencing risk from others in the local area or having expressed a preference to be placed away from home (Care Quality Commission, 2019). As Allen (Allen, 2008) reported, OAPs “would be redundant if local services were sufficiently competent to respond to the diverse and complex needs of people with learning disabilities,” (p. 5) suggesting that UK governments should create financial incentives for the development of local services and clearer financial consequences of placing individuals out-of-area. It should be acknowledged that local services do not necessarily guarantee quality; however, they do considerably reduce the likelihood of loneliness and social dislocation.

Aspects of clinical decision-making such as shared decision-making (Slade, 2017), accuracy (Miller et al., 2015), and the role of human factors (Heiden et al., 2017) have been limited within empirical analyses to how decisions are made in practice, although observational work has highlighted their influence on service user outcomes (Puschner et al., 2016) One study examined decision-making by MH crisis team clinicians (Lombardo et al., 2019); but limited research has focused upon the range of factors influencing practitioners’ decision-making in relation to OAPs for service users with CMH needs. There has been a paucity of research providing insight into the factors influencing clinician decision-making and the wider experience of treating individuals with complex needs; therefore, how clinicians define the experience of *providing* quality MH care for service users with CMH needs remains unclear. Overall, the clinicians appeared to engage in frank discussions about service shortcomings, stigma, variability in risk-taking behaviours within teams, and OAPs. They commented on what they *would* do when faced with complexity, rather than what they *should* do. Mostly, their views were in line with service users and carers. This level of feedback can inform the development of co-produced educational staff training to improve care; however, shared decision-making is part of a process of co-production and is not solely the clinician’s responsibility. Ideally, both service user and clinician should be trained in shared decision-making in order for it to make a real difference within the therapeutic relationship, as well as in the recovery of the person. In practice, a range of cultural, societal and system-level barriers exist which appear to hinder necessary attitudinal change from occurring among UK clinicians working with this population (Barned, 2016). Clinicians have been found to conceptualise shared decision-making differently to service users, with clinicians focusing on reducing clinical symptoms, whilst service users are more interested in participating in valued activities and achieving goals (Treichler et al., 2021). Robust back-and-forth conversations around decisions appear rare, possibly due to factors such as time constraints (Ramanuj & Pincus, 2019). Some clinicians have even reported excluding individuals with CMH needs from shared decision-making altogether (Treichler et al., 2021).

### Limitations

A strength of this research is the collection of in-depth data from individuals currently being supported by services. The focus was on high complexity, irrespective of diagnosis. Access to service users provides a closer review of reality, with the findings reflecting ‘real world’ service provision and the experiences of users of these services. The findings should be interpreted in the context of some methodological limitations, as the results may not be representative of the rest of the UK (as data were only collected in North-West England), although many of the issues identified are likely to apply across other areas. Another limitation is the lack of diversity, as most service users were White British (10/11), with limited participation from ethnic minority communities. It is important to note, however, that our numbers are largely representative of the ethnic background of the local community, with only 3.19% of Cheshire West and Chester residents classified as being from ethnic minority groups (Cheshire and Merseyside Health and Care Partnership, 2021). Despite this, we should aim to represent the wider population of the UK, of whom 14% are ethnic minorities (Bansal et al., 2022), and ensure all views are captured moving forward. Specific targeting of certain ethnic groups will aid future research and work to reduce mental healthcare disparities (Bansal et al., 2022).

### Conclusion

Through analysis of interviews in this study, the service user journey was found to be impacted by a range of factors, including relationships with staff, nature of support offered, financial constraints and organisational goals around bed pressures. These factors combine to influence the overall experience of care for service users and their carers. For some, this may contribute to positive treatment outcomes such as independent living, stability and recovery, and for others, negative treatment outcomes such as long-stay admissions, withdrawal from services and relapse. Recommendations included focusing on building therapeutic relationships, increasing autonomy and improving staff training. Initiatives such as NHS Choices and Patient Advice and Liaison Services (PALS) have highlighted the importance of considering service user experiences and improving these wherever possible (National Institute for Health and Care Excellence, 2011). However, service users with CMH needs have often been excluded from participating in service evaluation, despite their ability to offer useful insight (Chinn et al., 2011), as evidenced by our findings.

## Conclusions and recommendations

**Figure 4.**
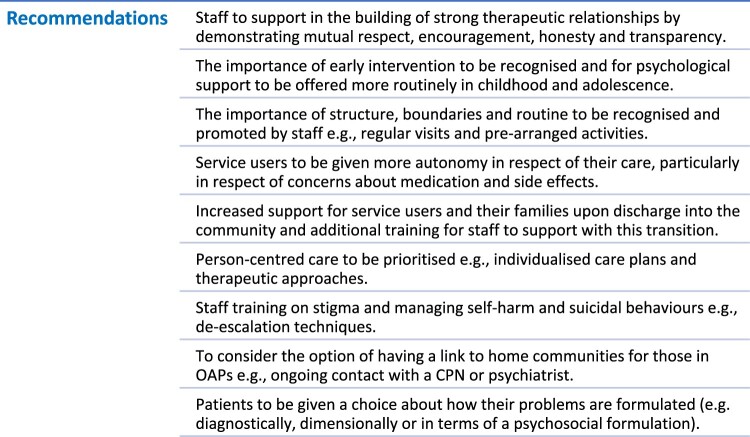
List of recommendations for implementation, based on participant interviews.

Recommendations that should be considered for implementation in future practice are highlighted in Figure 4.

## Supplementary Material

Supplemental Material

## Data Availability

The data that support the findings of this study are available from the corresponding author [L.M.Sambrook@ljmu.ac.uk], upon reasonable request.

## References

[CIT0001] Abou-Seif, N., Wood, L., & Morant, N. (2022). Invisible experts: A systematic review & thematic synthesis of informal carer experiences of inpatient mental health care. *BMC Psychiatry*, *22*(1), 347. 10.1186/s12888-022-03872-9 35596170 PMC9121622

[CIT0002] Allen, D. (2008). Failing to plan is planning to fail: Out of area placements for people. *Advances in Mental Health and Learning Disabilities*, *2*, 3–6.

[CIT0003] Bansal, N., Karlsen, S., Sashidharan, S. P., Cohen, R., Chew-Graham, C. A., & Malpass, A. (2022). Understanding ethnic inequalities in mental healthcare in the UK: A meta-ethnography. *PLoS Medicine*, *19*(12), 1–36. 10.1371/journal.pmed.1004139 PMC9746991 36512523

[CIT0004] Barned, N. L. (2016). Shared decision making and support for self-management: A rationale for change. *Future Healthcare Journal*, *3*(2), 117–120. 10.7861/futurehosp.3-2-117 PMC6465826 31098201

[CIT0005] Barrow, S., & Harrison, R. (2005). Unsung heroes who put their lives at risk? Informal caring, health and neighbourhood attachment. *Journal of Public Health*, *27*(3), 292–297. 10.1093/pubmed/fdi038 15985447

[CIT0006] Beadle-Brown, J., Mansell, J., Whelton, R., Hutchinson, A., & Skidmore, C. (2005). *Too far to go? People with learning disabilities placed out-of-area*. Tizard Centre.

[CIT0007] Bowen, M. (2013). Borderline personality disorder: clinicians’ accounts of good practice. *Journal of Psychiatric and Mental Health Nursing*, *20*(6), 491–498. 10.1111/j.1365-2850.2012.01943.x 22727023

[CIT0008] Bowers, L. (2005). *Dangerous and severe personality disorder: Reactions and role of the psychiatric team*. Routledge.

[CIT0009] Braun, V., & Clarke, V. (2006). Using thematic analysis in psychology. *Qualitative Research in Psychology*, *3*(2), 77–101. 10.1191/1478088706qp063oa

[CIT0010] Braun, V., & Clarke, V. (2019). Reflecting on reflexive thematic analysis. *Qualitative Research in Sport, Exercise and Health*, *11*(4), 589–597. 10.1080/2159676X.2019.1628806

[CIT0011] Care Quality Commission. (2014). *The state of care in mental health services 2014 to 2017.* Retrieved 5 March 2021. https://www.cqc.org.uk/publications/major-report/state-care-mental-health-services-2014-2017

[CIT0012] Care Quality Commission. (2019). *Brief guide: Out of area placements in rehabilitation units [online]*. Retrieved 5 February 2021. https://www.cqc.org.uk/sites/default/files/Brief_Guide_Out_of_Area_Placements_in_Rehabilitation_Units_0.pdf

[CIT0013] Cheshire and Merseyside Health and Care Partnership. (2021). *Ethnicity profiles in Cheshire and Merseyside.* Retrieved 9 May 2022. https://www.cheshireandmerseysidepartnership.co.uk/wp-content/uploads/2021/02/Ethnicity-profiles-in-Cheshire-Merseyside.pdf

[CIT0014] Chinn, D., Hall, I., Ali, A., Hassell, H., & Patkas, I. (2011). Psychiatric inpatients away from home: Accounts by people with intellectual disabilities in specialist hospitals outside their home localities. *Journal of Applied Research in Intellectual Disabilities*, *24*(1), 50–60. 10.1111/j.1468-3148.2010.00572.x

[CIT0015] Dalton-Locke, C., Marston, L., McPherson, P., & Killaspy, H. (2021). The effectiveness of mental health rehabilitation services: A systematic review and narrative synthesis. *Frontiers in Psychiatry*, *11*, 1–12. 10.3389/fpsyt.2020.607933 PMC7838487 33519552

[CIT0016] Farmer, T., Robinson, K., Elliott, S. J., & Eyles, J. (2006). Developing and implementing a triangulation protocol for qualitative health research. *Qualitative Health Research*, *16*(3), 377–394. 10.1177/1049732305285708 16449687

[CIT0017] Galante, J. R., Humphreys, R., & Molodynski, A. (2019). Out-of-area placements in acute mental health care: The outcomes. *Progress in Neurology and Psychiatry*, *23*(1), 28–30. 10.1002/pnp.528

[CIT0018] Gray, B., Robinson, C., Seddon, D., & Roberts, A. (2010). Patterns of exclusion of carers for people with mental health problems – the perspectives of professionals. *Journal of Social Work Practice*, *24*(4), 475–492. 10.1080/02650530903528821

[CIT0019] Heiden, S. M., Holden, R. J., Alder, C. A., Bodke, K., & Boustani, M. (2017). Human factors in mental healthcare: A work system analysis of a community-based program for older adults with depression and dementia. *Applied Ergonomics*, *64*, 27–40. 10.1016/j.apergo.2017.05.002 28610811 PMC5535802

[CIT0020] James, N. (2012). The formal support experiences of family carers of people with an intellectual disability who also display challenging behaviour and/or mental health issues: What do carers say? *Journal of Intellectual Disabilities*, *17*(1), 6–23. 10.1177/1744629512472610 23325117

[CIT0021] Killaspy, H. (2014). The ongoing need for local services for people with complex mental health problems. *The Psychiatric Bulletin*, *38*(6), 257–259. 10.1192/pb.bp.114.048470 25505623 PMC4248159

[CIT0022] Killaspy, H., & Zis, P. (2013). Predictors of outcomes for users of mental health rehabilitation services: A 5-year retrospective cohort study in inner London, UK. *Social Psychiatry and Psychiatric Epidemiology*, *48*(6), 1005–1012. doi:10.1007/s00127-012-0576-8 22945367

[CIT0023] Klevan, T., Karlsson, B., & Ruud, T. (2017). At the extremities of life – service user experiences of helpful help in mental health crises. *American Journal of Psychiatric Rehabilitation*, *20*(2), 87–105. 10.1080/15487768.2017.1302370

[CIT0024] Kon, A. A. (2010). The shared decision-making continuum. *JAMA*, *304*(8), 903–904. 10.1001/jama.2010.1208 20736477

[CIT0025] Krotofil, J., McPherson, P., & Killaspy, H. (2018). Service user experiences of specialist mental health supported accommodation: A systematic review of qualitative studies and narrative synthesis. *Health & Social Care in the Community*, *26*(6), 787–800. 10.1111/hsc.12570 29609195

[CIT0026] Lawn, S. (2015). Experiences of family carers of people diagnosed with borderline personality disorder. *Journal of Psychiatric and Mental Health Nursing*, *22*(4), 234–243. 10.1111/jpm.12193 25857849

[CIT0027] Leung, L. (2015). Validity, reliability, and generalizability in qualitative research. *Journal of Family Medicine and Primary Care*, *4*(3), 324. 10.4103/2249-4863.161306 PMC4535087 26288766

[CIT0028] Lombardo, C., Santos, M., Van Bortel, T., Croos, R., Arensman, E., & Kar Ray, M. (2019). Decision-making in crisis resolution and home treatment teams: The AWARE framework. *BJPsych Bulletin*, *43*(2), 61–66. 10.1192/bjb.2018.94 30451131 PMC6472319

[CIT0029] Magnavita, J. J. (2016). *Clinical decision making in mental health practice* (pp. xv–322). American Psychological Association.

[CIT0030] Maybery, D., Jaffe, I. C., Cuff, R., Duncan, Z., Grant, A., Kennelly, M., … Reupert, A. (2021). Mental health service engagement with family and carers: What practices are fundamental? *BMC Health Services Research*, *21*(1), 1–11. 10.1186/s12913-020-05996-8 34627245 PMC8502279

[CIT0031] Miller, K. E., Singh, H., Arnold, R., & Klein, G. (2020). Clinical decision-making in complex healthcare delivery systems. In J. E. Camacho-Cogollo, I. Bonet, & E. Iadanza (Eds.), *Clinical engineering handbook* (Second ed., pp. 858–864). Academic Press.

[CIT0032] Miller, D. J., Spengler, E. S., & Spengler, P. M. (2015). A meta-analysis of confidence and judgment accuracy in clinical decision making. *Journal of Counseling Psychology*, *62*(4), 553–567. 10.1037/cou0000105 26280710

[CIT0033] Mind. (2020). Mental health facts and statistics. Retrieved 5 September 2022. https://www.mind.org.uk/information-support/types-of-mental-health-problems/statistics-and-facts-about-mental-health/how-common-are-mental-health-problems/

[CIT0034] Muir-Cochrane, E., Gerace, A., Mosel, K., O’Kane, D., Barkway, P., Curren, D., & Oster, C. (2011). Managing risk: Clinical decision-making in mental health services. *Issues in Mental Health Nursing*, *32*(12), 726–734. 10.3109/01612840.2011.603880 22077745

[CIT0035] Nathan, R., Gabbay, M., Boyle, S., Elliott, P., Giebel, C., O’Loughlin, C., Wilson, P., & Saini, P. (2021). Use of acute psychiatric hospitalisation: A study of the factors influencing decisions to arrange acute admission to inpatient mental health facilities. *Frontiers in Psychiatry*, *12*, 1–9. 10.3389/fpsyt.2021.696478 PMC8273335 34262495

[CIT0036] The National Confidential Inquiry into Suicide and Safety in Mental Health. (2022). *Annual Report: UK patient and general population data, 2009-2019, and real time surveillance data*. University of Manchester.

[CIT0037] National Institute for Health and Care Excellence. (2011). Service user experience in adult mental health: Improving the experience of care for people using adult NHS mental health services. Retrieved 9 May 2022. https://www.nice.org.uk/guidance/cg136/resources/service-user-experience-in-adult-mental-health-improving-the-experience-of-care-for-people-using-adult-nhs-mental-health-services-pdf-35109513728197 31869024

[CIT0038] NHS England. (2019). Community mental health services. Retrieved 5 September 2022. https://www.england.nhs.uk/mental-health/adults/cmhs/

[CIT0039] Puschner, B., Becker, T., Mayer, B., Jordan, H., Maj, M., Fiorillo, A., … Slade, M. (2016). Clinical decision making and outcome in the routine care of people with severe mental illness across Europe (CEDAR). *Epidemiology And Psychiatric Sciences*, *25*(1), 69–79. 10.1017/S204579601400078X 25600424 PMC6998762

[CIT0040] Ramanuj, P. P., & Pincus, H. A. (2019). Collaborative care: Enough of the why; what about the how? *British Journal of Psychiatry*, *215*(4), 573–576. 10.1192/bjp.2019.99 31025616

[CIT0041] Rambarran, D. (2013). Relocating from out-of-area treatments: service users’ perspective. *Journal of Psychiatric and Mental Health Nursing*, *20*(8), 696–704. 10.1111/jpm.12003 22957942

[CIT0042] Rock, B., & Carrington, A. (2012). Complexity in primary care. In A. Lemma (Ed.), *Contemporary developments in adult and young adult therapy: The work of the Tavistock and Portman clinics*. (Vol. 1, pp. 111–133). Karnac Books.

[CIT0043] Ryan, T., Pearsall, A., Hatfield, B., & Poole, R. (2004). Long-term care for serious mental illness outside the NHS: A study of out of area placements. *Journal of Mental Health*, *13*(4), 425–429. 10.1080/09638230410001729861

[CIT0044] Slade, M. (2017). Implementing shared decision making in routine mental health care. *World Psychiatry*, *16*(2), 146–153. 10.1002/wps.20412 28498575 PMC5428178

[CIT0045] Stuart, R., Ferhana Akther, S., Machin, K., Persaud, K., Simpson, A., Johnson, S., & Oram, S. (2020). Carers’ experiences of involuntary admission under mental health legislation: Systematic review and qualitative meta-synthesis. *BJPsych Open*, *6*(19), 1–9. 10.1192/bjo.2019.101 PMC7176830 32043435

[CIT0046] Treichler, E. B. H., Rabin, B. A., Cohen, A. N., & Light, G. A. (2021). How shared is shared decision making? Reaching the full potential of patient-clinician collaboration in mental health. *Harvard Review of Psychiatry*, 1–9. 34352846 10.1097/HRP.0000000000000304 PMC12478557

