# Supplementary material for: The journey of service users with complex mental health needs: a qualitative study
Source: Health Psychol Behav Med. 2024 Jun 14;12(1):2365226. doi: 10.1080/21642850.2024.2365226 (PMC11182059; doi:10.1080/21642850.2024.2365226)
Supplement: Supplemental Material [file RHPB_A_2365226_SM2805.docx]

**Supplementary Materials**

**Appendix 1 - Service user interview schedule:**

You are being invited to take part in this project which is being conducted by Liverpool John Moores University and Cheshire and Wirral Partnership NHS Foundation Trust. The project aims to explore via 1:1 interviews with service users who have complex mental health needs. In addition the project aims to collate service user views on their experience of the management and treatment of their mental health condition.

Before you decide, it is important for you to understand why the project is being carried out and what it will involve. **Please take time to read the following information carefully and discuss it with others if you wish.** Ask us if there is anything that is not clear or if you would like more information. Take time to decide whether or not you wish to take part.

*Patients within this group from different types of placement and their carers will be approached to ask whether they are willing to speak to us about their experience of contact with relevant services and of the way decisions about their care were made.*

**Interview questions:**

1. Can you tell me about your experience of contact with mental health services?
2. What are your thoughts about the care you received for your mental health condition?
3. If you have been an inpatient, how did you find the decisions that were made in respect of your care?
   1. What was your involvement in the decision-making process?
   2. Did you experience a sense of autonomy in respect of your care? Why/why not?
   3. Could you tell me more about the psychological therapies you received?
   4. Were you prescribed psychotropic medication? Could you tell me more about your experience of this?
   5. Did you feel prepared to be discharged? Why/why not?
   6. How would you describe your relationships with staff? Did any of these relationships continue afterwards e.g. with social workers? Were these relationships consistent? Why/why not?
4. If you have been placed in any out of area placements, how did this affect you?
   1. Have you been in any local placements? If so, how did this affect you?
   2. If you were placed out of area, what was your understanding of why this was the case? Did you feel you were involved in the decision-making process? Did you feel it was the right decision for you? Why/why not?
   3. What helped and what was not as helpful in respect of the move?
5. Overall, in terms of your experience of contact with mental health services and the care you received, what helped and what was not as helpful?
6. In terms of positive experiences, why do you think these aspects were successful?
7. In terms of negative experiences, why do you think these aspects were unsuccessful? Were there placements that did not work? If so, why?
8. Do you have any suggestions about possible improvements that could be made?
9. Is there anything else you would like to discuss?

**Appendix 2 - Carer interview schedule:**

You are being invited to take part in this project which is being conducted by Liverpool John Moores University and Cheshire and Wirral Partnership NHS Foundation Trust. The project aims to explore via 1:1 interview with carers of service users with complex mental health needs who have been managed and treated within this Trust. In addition, the project aims to collate carers views on their experience of caring for patients with complex mental health needs.

Before you decide, it is important for you to understand why the project is being carried out and what it will involve. **Please take time to read the following information carefully and discuss it with others if you wish.** Ask us if there is anything that is not clear or if you would like more information. Take time to decide whether or not you wish to take part.

**Interview questions:**

1. **Can you tell me about your experience of caring for someone with complex mental health needs?**
   1. Once admitted, was the service provided as you expected?
   2. If the person you care for was admitted to the out-of-area placement, were you introduced the service?
   3. Was it explained to you what the placement looked like?
2. **When the person you care for was detained under the mental health act, how did you feel**?
   1. Were you involved in the decision for the person you care for to be detained under the mental health act?
   2. Was the person you care for happy for you to be involved in the decision?
3. **Were you asked your views about what led to the admission?**
   1. Were you asked for any family history?
   2. Were you asked about any events leading to the admission?
   3. Were you asked about any triggers that led to the person you care for being admitted?
4. **What are your thoughts about the care they have received for their mental health condition?**
   1. Were you involved in any of the decisions about their care?
   2. Was the person you care for happy for your involvement in their care?
   3. Is there anything you think could be improved?
5. **When the person you care for was admitted to hospital, how did this affect you?**

- Were there any issues when visiting them? What were they?

1. **If the person you care for was placed in out of area placements, how did this affect you?**
   1. Were there any issues when visiting them? What were they?

- Expensive
- Distance/Travel issues
- Ill health/disability to self
- Person you care for refused visits
  1. Was there a specific contact person in the ward?
- If yes, were they available 24/7?
  1. Was there any impact on the relationship between you and the person you care for?
  2. What were your experiences of visiting the person you care for, in an out of area placement for the first time?

1. **Is there anything that you think would’ve helped the person in not needing to be admitted to hospital?**
   1. Was the person you care for involved with community services?
   2. What do you think may have helped their mental health difficulties?
2. **Is there anything else you would like to discuss?**

**Appendix 3 - Clinician interview schedule:**

You are being invited to take part in this project which is being conducted by Liverpool John Moores University and Cheshire and Wirral Partnership NHS Foundation Trust. The project aims to explore via 1:1 interviews with clinicians in the decision-making processes used in the management of patients with complex mental health needs. In addition, the project aims to collate clinician views on their experience of the management and treatment of service users with complex mental health needs.

Before you decide, it is important for you to understand why the project is being carried out and what it will involve. **Please take time to read the following information carefully and discuss it with others if you wish.** Ask us if there is anything that is not clear or if you would like more information. Take time to decide whether you wish to take part.

**Interview questions:**

1. Can you tell me what you would do next in this circumstance?

*- Non-specific questions to explore what the clinician would do/the rationale for doing so. 
- Encourage participant to talk about the range of options they would consider and the reasons for ruling some out and preferring others.*

1. Do you have any thoughts about the care given to this patient so far? What are the issues that make this case more difficult to manage?

1. *Having encouraged the clinician to consider all options, if the participant does not mention an out-of-area placement, the interviewer should ask whether that is a consideration.*

Is this due to need and/or what’s available?

1. Do you have any experience of referring patients to out-of-area placements? If so, can you tell me about your experience?

*- Is this a positive or negative experience? 
- Is it straight-forward? 
- Overall, has it been of benefit to the patients?*

1. Do you feel clinical decisions are made with overall outcomes in mind?

1. Thinking about this case or other cases in your experience, how do you balance the long-term goals and immediate needs of the service user?

1. How confident would you be that community services would be able to meet the needs of a service user such as that in this scenario?

1. Is there anything else you would like to discuss?

**Appendix 4 - Diagram of themes and sub-themes:**
